# New Vaccine Introductions in WHO African Region between 2000 and 2022

**DOI:** 10.3390/vaccines11111722

**Published:** 2023-11-16

**Authors:** Chinwe Iwu-Jaja, Chidozie Declan Iwu, Anelisa Jaca, Charles Shey Wiysonge

**Affiliations:** 1 Communicable and Non-Communicable Diseases Cluster, World Health Organization Regional Office for Africa, Brazzaville P.O. Box 06, Congo; sheyc@who.int; 2 School of Health Systems and Public Health, Faculty of Health Sciences, University of Pretoria, Pretoria 0031, South Africa; chidoziedeclan@gmail.com; 3 Cochrane South Africa, South African Medical Research Council, Cape Town 7505, South Africa; anelisa.jaca@mrc.ac.za

**Keywords:** vaccine introduction, Africa, WHO, Gavi

## Abstract

Significant progress has been made in vaccine development worldwide. This study examined the WHO African Region’s vaccine introduction trends from 2000 to 2022, excluding COVID-19 vaccines. We extracted data on vaccine introductions from the WHO/UNICEF joint reporting form for 17 vaccines. We examined the frequency and percentages of vaccine introductions from 2000 to 2022, as well as between two specific time periods (2000–2010 and 2011–2022). We analysed Gavi eligible and ineligible countries separately and used a Chi-squared test to determine if vaccine introductions differed significantly. Three vaccines have been introduced in all 47 countries within the region: hepatitis B (HepB), *Haemophilus influenzae* type b (Hib), and inactivated polio vaccine (IPV). Between 2011 and 2022, HepB, Hib, IPV, the second dose of measles-containing vaccine (MCV2), and pneumococcal conjugate vaccine (PCV) were the five most frequently introduced vaccines. Hepatitis A vaccine has only been introduced in Mauritius, while Japanese encephalitis vaccine has not been introduced in any African country. Between 2000–2010 and 2011–2022, a statistically significant rise in the number of vaccine introductions was noted (*p* < 0.001) with a significant positive association between Gavi eligibility and vaccine introductions (*p* < 0.001). Significant progress has been made in the introduction of new vaccines between 2000 and 2022 in the WHO African Region, with notable introductions between 2011 and 2022. Commitments from countries, and establishing the infrastructure required for effective implementation, remain crucial.

## 1. Introduction

Vaccines are evidently one of the most cost-effective investments in health and development in history [1] that play a crucial role in preventing and controlling infectious diseases. They have led to significant reductions in morbidity and mortality for several decades [2,3], saving millions of lives yearly [4]. A recent study, which examined the impact of vaccination against 10 pathogens in 112 countries, estimated vaccination will save 97 million lives between 2000 and 2030, with 50 million lives saved between 2000 and 2019 [3]. The 10 pathogens included in the study were *Haemophilus influenzae* type b, hepatitis B, human papillomavirus, Japanese encephalitis, measles, *Neisseria meningitidis* serogroup A, rotavirus, rubella, *Streptococcus pneumoniae*, and yellow fever.

The Expanded Programme on Immunisation (EPI) was introduced in 1974, initially focusing on six diseases that could be prevented through vaccination. Since the year 2000, there has been a significant increase in the number of vaccines included in EPI schedules. Significant progress has been made in development, leading to licensed vaccines that effectively combat several globally [5]. In addition, immunisation programmes have expanded to include vaccinations for other age groups beyond infancy. This includes providing the second dose of measles-containing vaccines during the second year of life or later (MCV2), booster doses of DTP vaccine for preschool and school-age children, the introduction of HPV vaccines for preadolescents and adolescents, and the inclusion of seasonal influenza, pneumococcal, and herpes zoster vaccines for older adults. Most recently, COVID-19 vaccines have also been included in immunisation efforts.

In the past, there used to be a considerable delay in introducing new vaccines in lower-income countries compared to high-income countries. However, this time gap has been reduced, allowing for more equitable access to vaccines worldwide [6]. The introduction of new vaccines has been shown to bring about substantial improvements in a country’s health system [7]. Over the years, the World Health Organization (WHO) and the Global Alliance for Vaccines and Immunisation (Gavi) have made significant efforts to promote the introduction and utilisation of vaccines in various regions, including Africa, where the burden of vaccine-preventable diseases remains high [8]. The introduction of these vaccines has been a significant step towards reducing the burden of vaccine-preventable diseases in the region. The launch of the Global Vaccine Action Plan framework by WHO in 2012 aimed to prevent millions of deaths by 2020 through universal access to vaccines. This commitment has been further reinforced by the Immunisation Agenda 2030, which strives to ensure access to vaccines for everyone, including the importance of introducing new vaccines to address evolving health needs [9].

Despite the progress made, there are still challenges that need to be addressed to ensure these vaccines reach all those who need them. Expanding access to vaccines is critical for improving child health and survival [9]. Moreover, the majority of the sustainable development goals (SDGs) are directly impacted by immunization [10,11] The purpose of this study is to examine and describe the trends and patterns of vaccine introductions, except COVID-19 vaccines, in the WHO African Region between 2000 to 2022 since our focus was only on vaccines used for routine immunisations. We chose year 2000 as it coincides with the year when Gavi was established [12].

## 2. Methods

The data used in this study were accessed on 29 May 2023, from the WHO/UNICEF JRF [13]. The JRF dataset contains information on the introduction status of selected vaccines over time, categorised by country and WHO region. The introduction status is primarily classified as “Yes” or “No”, indicating whether the vaccine is administered in the country or not, respectively. Within the “Yes” category, there were additional sub-categories observed, including “Yes (P)” for vaccines administered in specific regions in a country, “Yes (R)” for vaccines targeting specific risk groups, and “Yes (D)” which was undefined in the dataset. For the analyses, we considered all instances of “Yes” regardless of the sub-categories.

A total of 17 vaccines which were found in the dataset were all included in the analysis, namely: acellular pertussis vaccine, hepatitis B vaccine (Hep B), hepatitis B vaccine given at birth (Hep B birth dose), *Haemophilus influenzae* type b containing vaccine (Hib), pneumococcal conjugate vaccine (PCV), the second dose of the measles-containing vaccine (MCV 2), meningococcal meningitis vaccine, rubella vaccine, mumps vaccine, yellow fever (YF) vaccine, Japanese encephalitis (JE) vaccine, rotavirus vaccine, human papillomavirus vaccine (HPV), inactivated polio-containing vaccine (IPV), second dose IPV (IPV2), and seasonal influenza vaccine.

We described the frequency and percentages of vaccine introductions during the entire study period (2000–2022) as well as between two time periods (2000–2010 and 2011–2022). We chose to divide these years to represent the time before and after the ‘Decade of Vaccines,’ which was launched in 2010 and lasted from 2010 to 2020. This strategy was part of the Global Vaccine Action Plan (GVAP), which preceded the Immunisation Agenda 2030 (IA2030) [14]. Additionally, we analysed the vaccine introductions individually for Gavi eligible and ineligible countries [15] within the two time periods. Based on the observed frequencies, Chi-squared test was conducted to determine if there were significant differences in vaccine introductions over the entire study period. We also assessed the relationship between Gavi eligibility and vaccine introductions within the two time periods. Furthermore, individual Chi-square tests (or Fisher’s exact tests where necessary) were performed for each vaccine to explore the association between Gavi eligibility and the introduction of specific vaccines within the two time periods. The analyses and visualisations were conducted using R Studio (version 4.2.1) and Microsoft Excel 2010, respectively. A *p*-value of 0.05 or less was considered to be statistically significant. To account for any chance of making a Type I error due to multiple comparisons conducted in this study, we performed a Bonferroni correction to the significance levels for each test [16,17].

## 3. Results

### 3.1. Number of Countries That Have Introduced Each Vaccine

**Figure 1:**
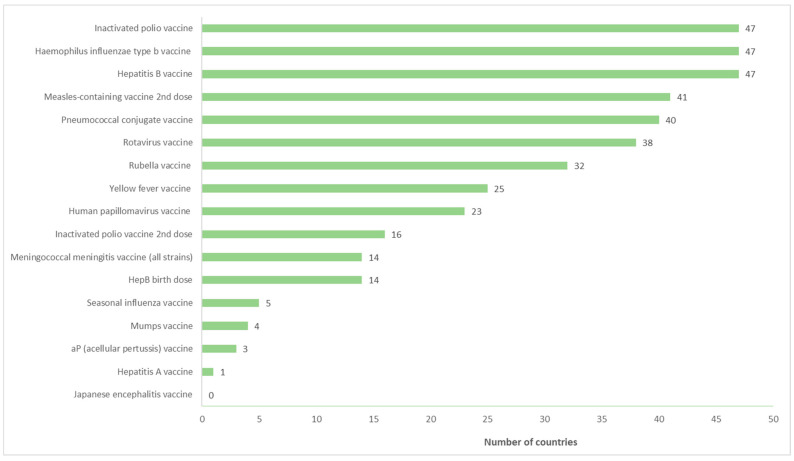
Number of countries which have introduced each vaccine between 2000 to 2022.

Our analysis reveals the following findings regarding the introduction of various vaccines across different countries in the WHO African Region as shown in Figure 1. Each of the 47 countries introduced at least one new vaccine between 2000 and 2022. Hepatitis B vaccine, Hib, and IPV vaccines have been introduced in all 47 countries. Other vaccines that have shown substantial introductions include MCV2, introduced in 41 countries, while PCV and rotavirus vaccines have been introduced in 40 (85%) and 38 (81%) countries, respectively. The second dose of IPV has been introduced in 16 (34%) countries. Forty-five countries introduced the HepB vaccine between 2000 and 2010, and by 2014, all 47 countries had introduced it. Of these 47 countries, 37 of them are Gavi eligible, while 10 are Gavi ineligible (Table 1).

The introduction of the hepatitis B birth dose vaccine, on the other hand, showed a noticeable increase after 2012, continuing until 2022. However, before then, no significant increases were observed from 2004 to 2010 as the number of countries introducing this vaccine only rose from two in 2001 to six in 2010. As of 2022, nine Gavi eligible countries and five non-Gavi eligible countries have introduced the HepB birth dose vaccine.

Furthermore, there was a significant increase in the number of countries introducing the Hib vaccine between 2007 and 2010, rising from 14 to 40 countries. By 2019, all 47 countries had introduced the Hib vaccine. These countries include 37 Gavi eligible and 10 Gavi ineligible countries.

Our findings also highlight the HPV, meningococcal meningitis, mumps, rubella, and yellow fever vaccines have been introduced in a varying number of countries, ranging from 23 to 32. In contrast, the seasonal influenza vaccine has only been introduced in three countries, and the mumps vaccine in four countries. Additionally, the introduction of the acellular pertussis (aP) and the hepatitis A vaccines lags behind, with only three and one countries respectively having introduced them. The Japanese encephalitis vaccine has not been introduced in any country.

The HPV vaccine was first introduced in 2011, and by 2019, a total of 16 countries had introduced it. As of 2022, the number of countries that introduced the HPV vaccine increased to 23, including 18 Gavi eligible and five Gavi ineligible countries. One country had introduced the IPV in 2009, but a significant leap in introductions was observed by 2015 when 26 countries had introduced it. By 2022, all 47 countries had introduced at least one dose of the IPV, which includes 37 Gavi eligible and 10 Gavi ineligible countries.

Similar to the IPV, only one country introduced the second dose of IPV vaccine in 2009; however, as of 2022, 16 countries had introduced this vaccine. Fourteen of these countries are Gavi eligible and two are Gavi ineligible.

Five countries introduced the second dose of the measles-containing vaccine by 2010. Subsequently, an increasing trend was observed, with 11 countries introducing it in 2012. By 2022, the majority of countries (*n* = 41) had introduced this vaccine. Of these forty-one countries, thirty-two were from Gavi eligible countries and nine from Gavi ineligible countries. The first introduction of the meningococcal vaccine occurred in 2008 in one country, which remained unchanged until 2010. A notable increase was observed in 2018 when seven countries introduced this vaccine. As of 2022, a total of 14 countries had introduced it, including 13 Gavi eligible countries and one Gavi ineligible country. Two countries introduced the mumps vaccine in 2000, and as of 2022, only four countries have introduced it. These countries are all Gavi ineligible. Forty countries have currently introduced the PCV as of 2022, and of these, thirty-three are Gavi eligible while seven are Gavi ineligible. The rotavirus vaccine was first introduced in one country in 2008, and by 2013, twelve countries had introduced it. By 2022, a total of thirty-eight countries in the region had introduced the rotavirus vaccine, which includes thirty-two Gavi eligible and two Gavi ineligible countries. As of 2022, the seasonal influenza vaccine has been introduced in only five countries since its initial introduction by one country in 2008. Four of these countries are Gavi eligible countries, and only one is a Gavi eligible country. In 2000, 12 countries had introduced the yellow fever vaccine, which increased significantly to 22 countries in 2004. However, the frequency of introduction plateaued, with 25 countries introducing it in 2022. Of these 25 countries, the majority of (*n* = 21) are Gavi eligible while four are Gavi ineligible. The acellular pertussis vaccine has been introduced in only two countries since 2000. Similarly, hepatitis A has been introduced in one country, which is a Gavi ineligible country.

**Figure 2:**
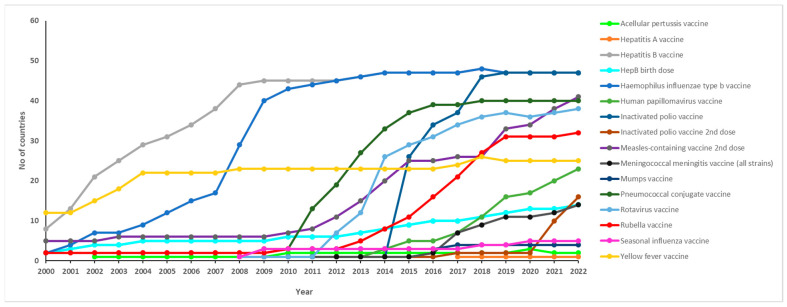
Trends in new vaccine introductions per year in the WHO African Region from 2000 to 2022.

Supplementary Table S1 summarises the number of Gavi eligible and ineligible countries that have introduced each new vaccine. Figure 2 shows the trend in vaccine introductions between 2000 and 2022.

### 3.2. Summary of Vaccine Introductions 2000 to 2010 vs. 2011 to 2022

**Figure 3:**
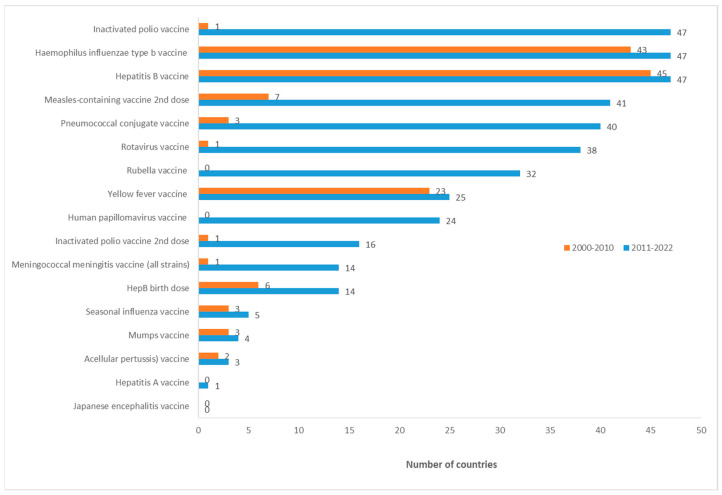
Summary of vaccine introductions 2000 to 2010 vs 2011 to 2022 in the WHO African Region.

Between 2000 and 2010, the top five most frequently introduced vaccines were HepB, Hib, yellow fever, MCV2, and HepB birth dose vaccines in decreasing order (Figure 3). Additionally, the top five most frequently introduced vaccines between year 2011 and 2022 were HepB, Hib, IPV, MCV2, and PCV vaccines in decreasing order (Figure 3). Based on the results of the chi-squared test, a significant increase was observed in the number of countries that introduced vaccines overall between the time periods of 2000–2010 and 2011–2022 (*p* < 0.001).

### 3.3. Country Level Vaccine Introduction between 2000 and 2022

All 47 countries have introduced the hepatitis B, Hib, and IPV vaccines. The hepatitis B birth dose vaccine has been introduced in 14 countries, namely Angola, Benin, Burkina Faso, Botswana, Côte d’Ivoire, Cabo Verde, Algeria, Gambia, Equatorial Guinea, Mauritania, Namibia, Nigeria, Senegal, and Sao Tome and Principe. Similarly, the HPV vaccine has been introduced in half of the countries in the WHO African region (*n* = 24), including Angola, Burkina Faso, Botswana, Côte d’Ivoire, Cameroon, Cabo Verde, Eritrea, Mauritius, Malawi, Rwanda, Senegal, Sierra Leone, Sao Tome and Principe, Seychelles, United Republic of Tanzania, Uganda, South Africa, Zambia, and Zimbabwe.

The yellow fever vaccine has been introduced in 25 countries, including Angola, Benin, Burkina Faso, Central African Republic, Côte d’Ivoire, Cameroon, Democratic Republic of the Congo, Congo, Cabo Verde, Mali, Niger, Nigeria, Senegal, Sierra Leone, Sao Tome and Principe, Seychelles, and Chad. Furthermore, PCV has been introduced in the majority of countries, except Comoros, Cabo Verde, Gabon, Guinea, Equatorial Guinea, South Sudan, and Chad. Similarly, the rotavirus vaccine has been introduced in the majority of countries, except Central African Republic, Comoros, Cabo Verde, Algeria, Gabon, Guinea, Equatorial Guinea, South Sudan, and Chad.

Meningococcal vaccines have been introduced in 14 countries, namely Burkina Faso, Central African Republic, Côte d’Ivoire, Eritrea, Ghana, Guinea, Gambia, Mali, Mauritius, Niger, Nigeria, Chad, and Togo. Moreover, the second dose of IPV has been introduced in 16 countries, namely Angola, Burkina Faso, Democratic Republic of Congo, Eritrea, Gambia, Madagascar, Mali, Mauritius, Niger, Nigeria, Sierra Leone, South Sudan, Chad, Togo, South Africa, and Zimbabwe. Additionally, the second dose of the measles-containing vaccine has been introduced in all countries except Benin, Central African Republic, Gabon, Mauritania, South Sudan, and Uganda. In Nigeria, it was introduced only in specific regions.

The acellular pertussis vaccine has only been introduced in three countries, namely Burkina Faso, Mauritius, and South Africa. Similarly, the mumps vaccine has been introduced in four countries, namely Cabo Verde, Algeria, Mauritius, and Seychelles. Additionally, the seasonal influenza vaccine has been introduced in five countries, namely Côte d’Ivoire, Algeria, Mauritius, Namibia, and South Africa.

On the other hand, the rubella vaccine has been introduced in the majority of countries, except in Central African Republic, Chad, Democratic Republic of the Congo, Ethiopia, Gabon, Guinea, Guinea-Bissau, Equatorial Guinea, Liberia, Madagascar, Mali, Niger, Nigeria, South Africa, and South Sudan. Furthermore, the hepatitis A vaccine has only been introduced in one country, Mauritius. Lastly, it is worth noting the Japanese encephalitis vaccine has not been introduced in any country.

Supplementary Figures S1–S16 show maps of Africa depicting distribution of vaccine introductions in WHO African region as of May 2022, namely, HepB, Hib, IPV, MCV2, PCV, Rotavirus, Rubella, Yellow fever, HPV, IPV2, meningococcal meningitis, HepB birthdose, seasonal influenza, mumps, acellular pertussis, HepA vaccines. 

### 3.4. Relationship between Gavi Eligibility and Vaccine Introductions

In the year 2000, there were 36 Gavi eligible countries, which increased to 37 in 2022. The number of Gavi ineligible countries was nine in the period of 2000–2010 and ten in the period of 2011–2022. The chi-square test was conducted to investigate the relationship between Gavi eligibility (Gavi eligible vs. Gavi ineligible) and vaccine introductions across the entire period of 2000–2022. Based on the test results, there was a significant difference between Gavi eligibility and vaccine introductions across the entire study period, with Gavi eligible countries introducing more vaccines (*p* < 0.001). However, for the time period of 2000–2010, no significant difference was found between Gavi eligibility and vaccine introductions in both Gavi eligible and Gavi ineligible countries (*p* > 0.05). The same analysis was repeated for the time period of 2011–2022, and again, no significant association was found (*p* = 1).

However, when analysing individual vaccines, no significant difference was found between the proportion of vaccine introductions in Gavi eligible and Gavi ineligible countries for all vaccines except Meningococcal meningitis vaccines (all strains) (*p* < 0.001).

## 4. Discussion

This study was aimed at describing the introduction status of new vaccines over time, per country within the WHO African Region. Our findings show every country in the WHO African region has introduced at least one new vaccine during the study period. Notably, three vaccines have been introduced in all 47 countries within the region, i.e., the Hep B, Hib, and the IPV vaccines. Additionally, some vaccines were introduced at accelerated rate between 2011 to 2022 including Hep B, Hib, IPV, MCV2, PCV, conjugate rubella, and rotavirus vaccines. The widespread introduction of the IPV vaccine, which protects against polio, particularly the vaccine-derived polio, also signifies the region’s commitment to eradicating polio and ensuring population immunity. Although the second dose of IPV has been introduced in a more limited number of countries, its introduction demonstrates the ongoing efforts to enhance immunisation programs and strengthen protection against polio.

These significant increases in the number of countries that introduced vaccines in the last decade are also reflected in the report and lessons from the Global Vaccines Action Plan (GVAP) [14]. This significant introduction in this region can be attributed to global commitments to achieving set goals and the strengthening technical and resource capacities of low-income and middle-income countries [5]. Additionally, the initiatives focused on polio eradication are thought to have played a significant role in driving increased vaccine introductions in the region [18].

Our study revealed there were no significant disparities in the introduction of new and underutilized vaccines between Gavi eligible and Gavi ineligible countries during the study period. These findings align with our previous study, which examined vaccine introductions from 2000 to 2017 [19]. Based on these findings on the association between Gavi eligibility and vaccine, Gavi eligibility had a significant association with overall vaccine introductions across the entire study period. However, when analyzing specific time periods and individual vaccines, other factors may have played a role in determining vaccine introduction patterns. The results emphasize the complex interplay of various factors influencing vaccine introductions and highlight the importance of considering context-specific dynamics when examining the impact of Gavi support. Recent developments from Gavi are underway. Countries will now have an opportunity to request funding as a window for applications is set to open. The introduction of the hexavalent vaccine is anticipated to enhance the delivery of protection against multiple diseases in a more efficient and cost-effective manner. Furthermore, it is expected to play a significant role in global polio eradication efforts by expanding the coverage of IPV [20].

Several studies conducted in WHO African region have documented valuable insights and benefits from vaccine introductions. These studies consistently show a reduction in disease burden and mortality highlighting the importance and positive outcomes associated with vaccine introductions. For instance, a study investigated the impact of rotavirus vaccines in the WHO African region by analysing the number of hospitalizations before and after their introduction. Between 2008 and 2018, countries that had introduced the vaccine experienced a significant decrease in overall hospitalizations, as well as within specific sub-regions. In contrast, countries that had not implemented the vaccines during the study period saw unchanged hospitalization rates [21]. A number of other African countries have conducted studies to assess the effects of routine rotavirus vaccination on the decrease in deaths and hospitalizations caused by rotavirus diarrhoea [22]. These studies were conducted in South Africa [23], Ghana [24], Malawi [25], Rwanda [26], Kenya [27], Mozambique [28], including a global study [29].Similarly, the introduction of the Hib vaccine in South Africa, Kenya, and Mozambique resulted in a notable decrease in Hib disease burden in these countries [30,31,32]. Furthermore, the impact of PCV introduction in South Africa, as examined by Madhi and Nunes, indicated reduced disease burden [33]. These public health gains are predicted to increase in coming decades if progress in vaccination coverage is sustained [2]. Since its recommendation in 1992, the hepatitis B vaccine has been progressively introduced in all countries within the WHO African Region over a span of 30 years [5]. This introduction has contributed to significant reductions in hepatitis B incidence within the region, which could be attributed to its inclusion in combination vaccines [34]. From 1990 to 2019, the all-age prevalence of chronic HBV decreased from 10% to 7.5% across the WHO African region, and this decline continued from 2015 to 2019 [35]. To combat the spread of hepatitis B, the World Health Organization has set a global target for its eradication by 2030 [34]. The HPV vaccine has been introduced in approximately half (51%) of the 47 countries in the WHO African region. The HPV vaccine was first introduced in 2013, and there has been a remarkable increase in the number of countries adopting it since then, with the number rising from one country in 2013 to 23 countries in 2022, which is quite impressive. The WHO recommends PCV be included in all global immunisation systems because pneumonia caused by various strains of the bacteria pneumococcus is associated with high morbidity and mortality, particularly in children under the age of five [36]. The introduction of PCV has significantly reduced the incidence of childhood pneumonia, including hospitalisations, and also burden in other children and adults who have not been vaccinated against it [37].

Although some progress has been made in the region, many disease elimination targets have not yet been reached, with vaccination coverage remaining at sub-optimal levels [38]. The WHO recommended rotavirus vaccination be included in all national immunisation schedules [39]. This is especially important in regions including Africa, where rotavirus gastroenteritis has a high mortality rate [39]. While the impact of vaccine introduction has shown decline in the incidence of the disease globally, Africa continues to disproportionately shoulder the impact, accounting for around 80% of the worldwide burden of rotavirus mortality [22]. Also, WHO emphasised the importance of preventing mother-to-child transmission of hepatitis B virus (HBV) by recommending universal hepatitis B birth dose (HepB-BD) vaccination, even in countries with low HBV prevalence [40]. However, many countries in the WHO African region have yet to introduce HepB-BD. The need for more countries in the region to introduce HepB-BD is crucial.

Despite available resources from donors to introduce vaccines in countries, the decision to introduce vaccines typically relies on several factors, including disease burden, public health priority, economic considerations, supply chain infrastructure, and readiness for implementation [7]. Other factors may include lack of disease burden data or vaccine hesitancy by the community, health system, or policymakers [5] and in some cases, global shortages [6]. For example, the global switch from oral polio vaccine to IPV was a significant achievement for the polio programme. However, after 2016, the programme faced various challenges. These included global shortages of IPV, which resulted in lower vaccination coverage in countries where it was introduced, as well as delays in IPV introduction in many countries [6]. Furthermore, despite Gavi’s endeavour to expedite the rollout of vaccines in low-income countries, significant financial obstacles could persist for countries that fail to meet the eligibility criteria for Gavi funding, yet still face financial constraints in affording new vaccines. For instance, certain middle-income countries, which were never eligible for Gavi support, are also lagging behind in introducing specific vaccines compared to Gavi eligible countries. While these vaccines may be accessible in these countries, they are often limited to the private sector, rendering them unavailable to vulnerable populations. An example of this is the Rubella vaccine in South Africa. To address these challenges, and interestingly so, Gavi has adopted a new strategic approach that aims to promote the introduction of new vaccines in Gavi ineligible countries and prevent a decrease in immunisation coverage in countries that have transitioned out of Gavi [41]. The delay in introducing the vaccine in certain countries could be attributed to concerns related to the vaccine itself, such as the paradoxical increase in congenital rubella cases observed in countries with insufficient coverage [6].

These challenges highlights the ongoing need for more vaccine introductions while emphasizing the importance of addressing other factors to ensure universal access to these vaccines [38]. These interventions have been highlighted in the regional plan aimed at achieving the 2030 immunisation agenda, which includes health system strengthening, effective political will, improving service delivery to reach children who have not received any vaccines or are under-immunised, and enhancing data systems to support informed decision-making and also strengthening immunisation systems to withstand challenges and enhancing capacity in vaccine logistics [38]. Additionally, strong surveillance systems and monitoring the impact of vaccine impact and effectiveness in the short- and long-term are critical in making vaccine introduction decisions [42], which is also in line with the 2030 immunisation agenda [43].

Additionally, it is important to acknowledge the COVID-19 pandemic may have had an impact on the rate at which new vaccines were introduced, especially its association with the access to vaccines and disruption of essential health services. Between 2020 and 2022, there were relatively few new vaccine introductions due to the disruptions caused by the pandemic. However, as we gradually recover from the effects of the pandemic, a renewed momentum will be crucial in contributing to the fulfilment of the new vision and impact goals outlined in the Immunization Agenda 2030 (IA2030) and ultimately help achieve the Sustainable Development Goals by the year 2030.

Furthermore, it is important to acknowledge the mere inclusion of select vaccines in national immunization schedules reported by countries does not necessarily guarantee children are effectively receiving these vaccinations. It is worth noting vaccine introduction comes with challenges that require consideration, especially as newer and improved vaccines such as those against meningitis, cholera, typhoid, malaria, and dengue will make their way into national immunisation schedules in the future. Insufficient vaccination coverage may lead to missed opportunities for vaccination. Therefore, it is important for efforts to be made to ensure the benefits of these introductions are maximized. There is also a need for strong commitment and financial support from countries, regardless of Gavi eligibility, coupled with proper guidelines to guide the process [5].

Our study was limited to only vaccines used in routine immunisations in countries within the WHO African region. Also, based on our data source, the newer vaccines such as COVID-19, typhoid, and malaria vaccines were not included in our study. Additionally, our analyses were also mostly descriptive, providing a comprehensive overview of new vaccine introductions, though without advanced statistical analysis performed. We intend to present our findings to relevant decision-makers and stakeholders seeking their opinions on the relevant indicators to include in future iteration of our analysis.

In summary, new vaccine introduction brings significant benefits by strengthening a country’s immunisation programme and overall health system [5]. There are several other factors that need to be considered to overcome barriers to vaccine adoption. These include enhancing communication efforts to raise awareness about the essential factors required for making evidence-based decisions that align with a country’s health objectives, conducting research activities to address critical questions that support the introduction of vaccines and ensure the long-term sustainability of vaccination programmes, coordinating with stakeholders at the global, regional, and country levels to effectively implement vaccination programmes and ensure their success [44].

## 5. Conclusions

During the period from 2000 to 2022, notable advancements have been made in new vaccine introductions, particularly in the past decade, indicating substantial progress. These findings emphasize the importance of integrating vaccines into immunisation programmes in countries where they have not been introduced. There are still considerable tasks ahead. These include securing sufficient funding through mechanisms like Gavi, ensuring commitments from countries, and establishing the necessary infrastructure for effective implementation. These are crucial to sustain efforts aimed at introducing additional vaccines in the remaining countries of the region and enhancing vaccine coverage to maximize the public health benefits provided by available vaccines.

## Figures and Tables

**Table 1:** Association between Gavi eligibility and vaccine introductions.

	2000–2010					2011–2022				
Vaccine	Gavi Eligible Countries (*n* = 36)	%	Gavi Ineligible Countries (*n* = 9)	%	*p*-Value *	Gavi-Eligible Countries (*n* = 37)	%	Gavi-Ineligible Countries (*n* = 10)	%	*p*-Value *
Acellular pertussis vaccine	0	0	2	22.2	-	1	2.70	2	20	1.00
Hep A vaccine	0	0	0	0.0	-	0	0	1	10	1.00
Hep B vaccine	36	100	9	100	0.27	37	100	10	100	1.00
Hep B birth dose	3	8.3	3	33.3	0.12	9	24.3	5	50	0.61
Hib	35	97.2	8	88.9	1.00	37	100	10	100	0.99
HPV	0	0	0	0	-	19	51.4	5	50	1.00
IPV	0	0	1	11.1	-	37	100	10	100	0.92
IPV2	23	63.9	1	11.1	-	14	37.8	2	20	1.00
MCV2	1	2.8	6	66.7	<0.001	32	86.5	9	90	1.00
Meningococcal meningitis vaccines	0	0	1	11.1	-	13	35.4	1	10	<0.001
Mumps vaccine	0	0	3	33.3	-	0	0	4	40	1.00
PCV	2	5.6	1	11.1	0.87	33	89.2	7	70	1.00
Rotavirus vaccine	0	0	1	11.1	-	32	86.5	6	60	1.00
Rubella vaccine	0	0	3	33.3	-	25	67.6	7	70	1.00
Seasonal influenza vaccine	0	0	3	33.3	-	1	2.7	4	40	1.00
Yellow fever vaccine	21	58.3	2	22.2	0.66	21	56.7	4	40	1.00
Japanese Encephalitis	0	0	0	0	-	0	0	0	0	-

* The significance level using the Bonferroni corrections established at *p* = 0.003.

## Data Availability

Data used for this study were publicly available.

## Supplementary Materials

The following supporting information can be downloaded at: https://www.mdpi.com/article/10.3390/vaccines11111722/s1, Table S1: Summarises the number of Gavi eligible and ineligible countries that have introduced each new vaccine. Figure S1: Status of Hepatitis B vaccine introduction in the WHO African region as of May 2022. Figure S2: Status of Hib vaccine introduction in the WHO African region as of May 2022. Figure S3: Status of IPV introduction in the WHO African region as of May 2022. Figure S4: Status of MCV2 introduction in the WHO African region as of May 2022. Figure S5: Status of PCV introduction in the WHO African region as of May 2022. Figure S6: Status of rotavirus vaccine introduction in the WHO African region as of May 2022. Figure S7: Status of rubella vaccine introduction in the WHO African region as of May 2022. Figure S8: Status of yellow fever vaccine introduction in the WHO African region as of May 2022. Figure S9: Status of HPV vaccine introduction in the WHO African region as of May 2022. Figure S10: Status of IPV2 introduction in the WHO African region as of May 2022. Figure S11: Status of meningococcal meningitis vaccine introduction in the WHO African region as of May 2022. Figure S12: Status of HepB birth dose vaccine introduction in the WHO African region as of May 2022. Figure S13: Status of seasonal influenza vaccine introduction in the WHO African region as of May 2022. Figure S14: Status of mumps vaccine introduction in the WHO African region as of May 2022. Figure S15: Status of acellular vaccine introduction in the WHO African region as of May 2022. Figure S16: Status of hepatitis A vaccine introduction in the WHO African region as of May 2022.
