# Supplementary material for: New Vaccine Introductions in WHO African Region between 2000 and 2022
Source: Vaccines (Basel). 2023 Nov 16;11(11):1722. doi: 10.3390/vaccines11111722 (PMC10675678; doi:10.3390/vaccines11111722)
Supplement: Supplementary file 1 [file vaccines-11-01722-s001.zip › vaccines-2651925-supplementary figures.pdf]

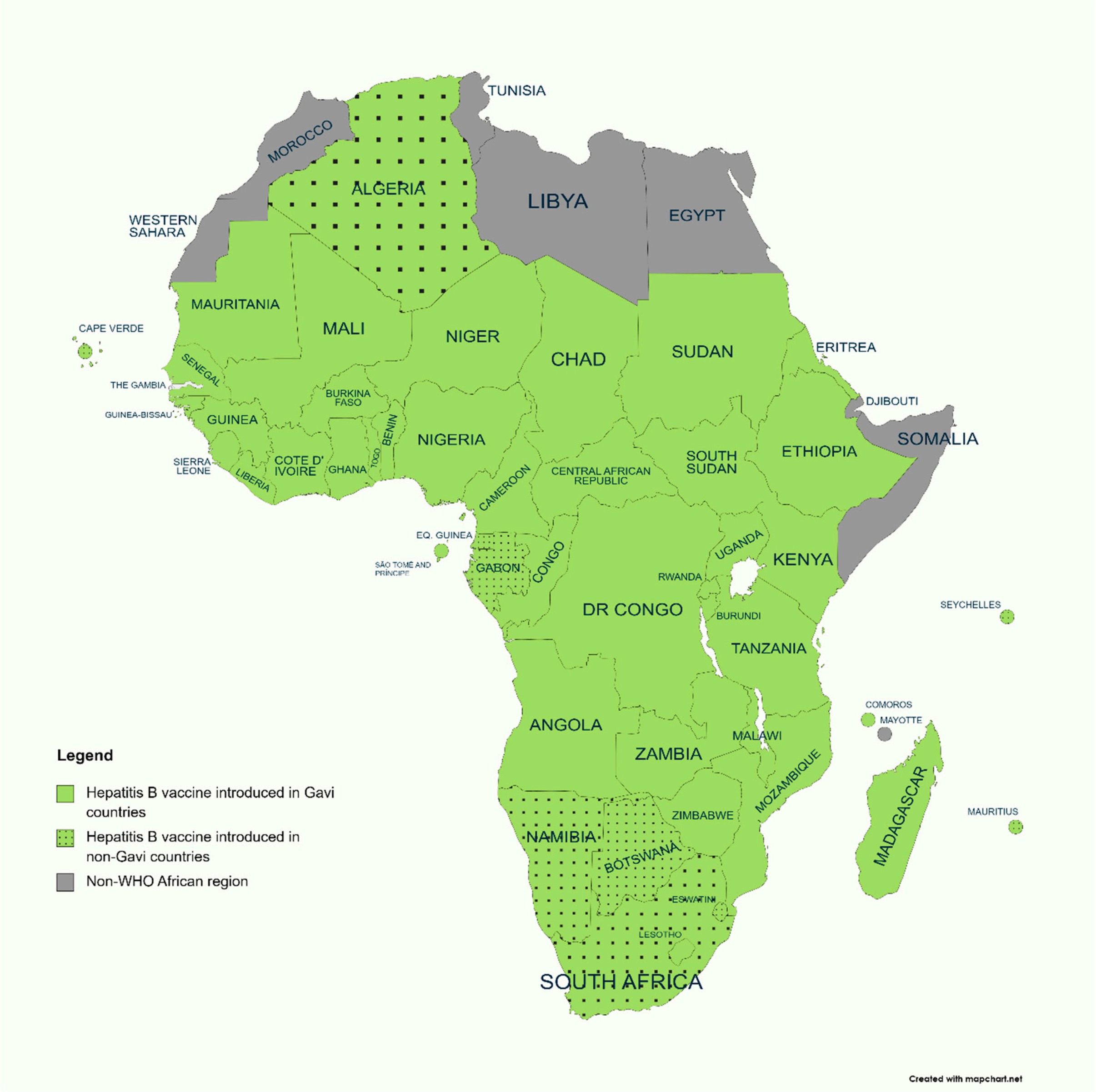

Figure S1. Status of Hepatitis B vaccine introduction in the WHO African region as of May 2022.

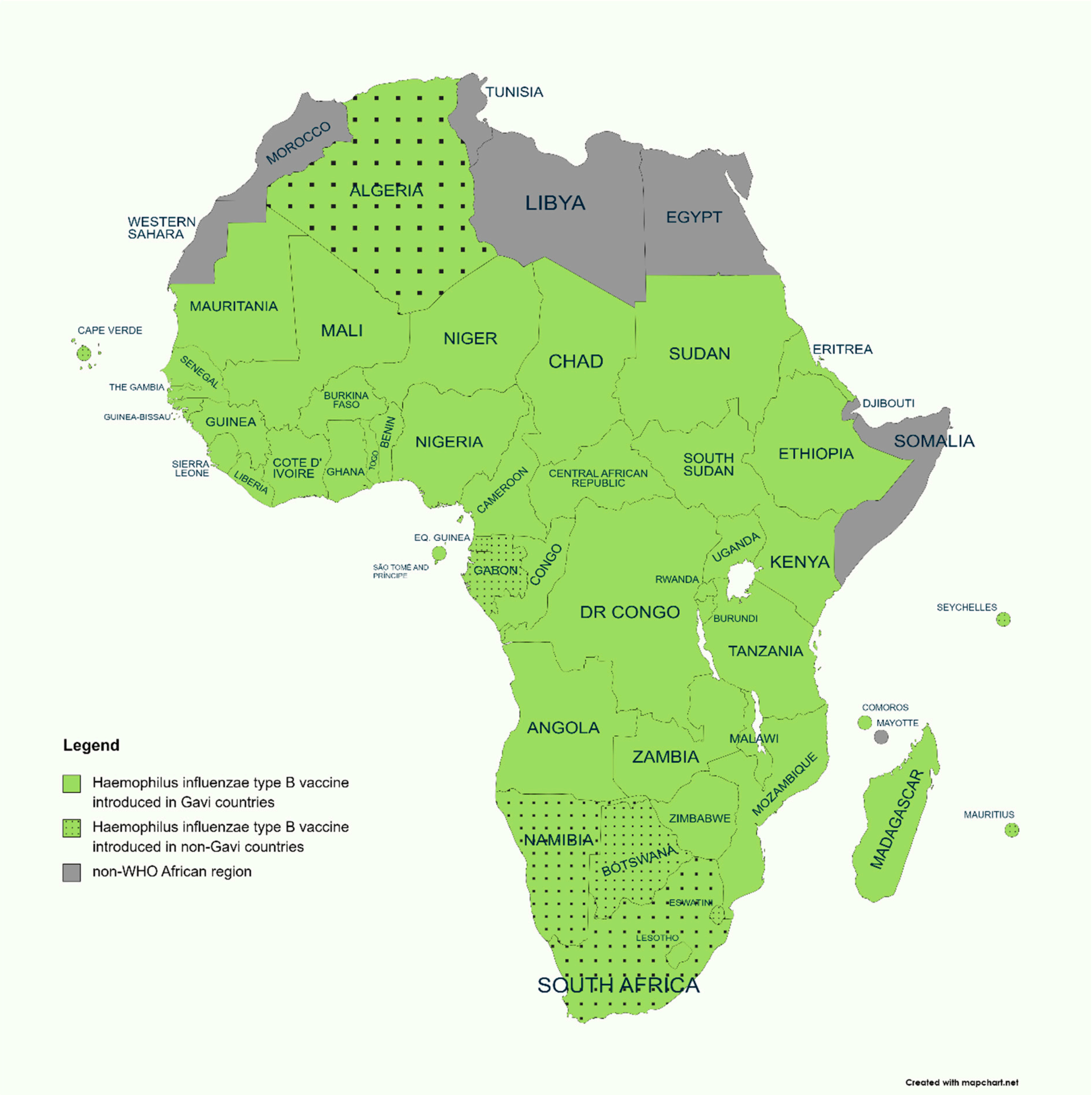

Figure S2. Status of Hib vaccine introduction in the WHO African region as of May 2022.

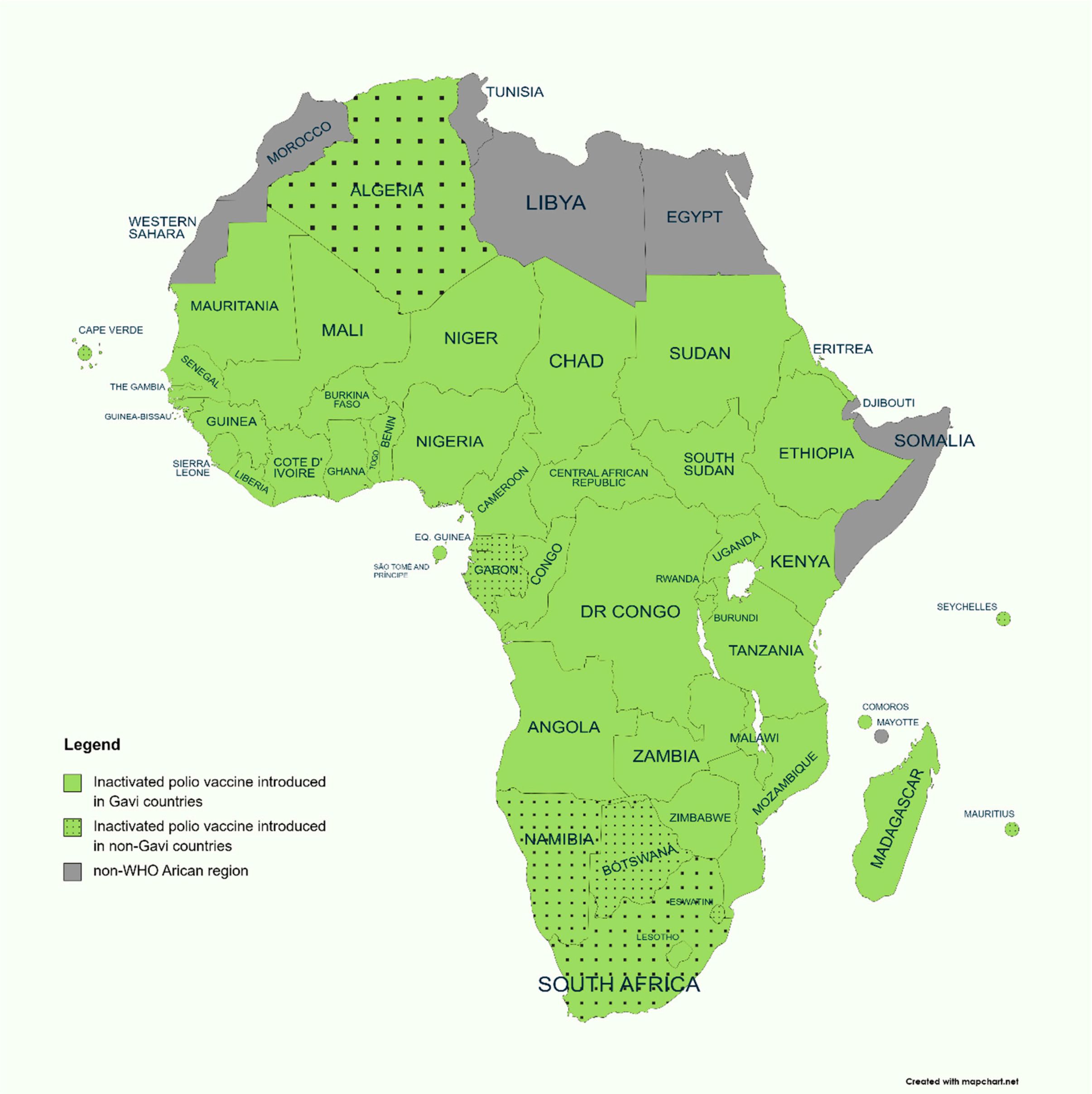

Figure S3. Status of IPV introduction in the WHO African region as of May 2022.

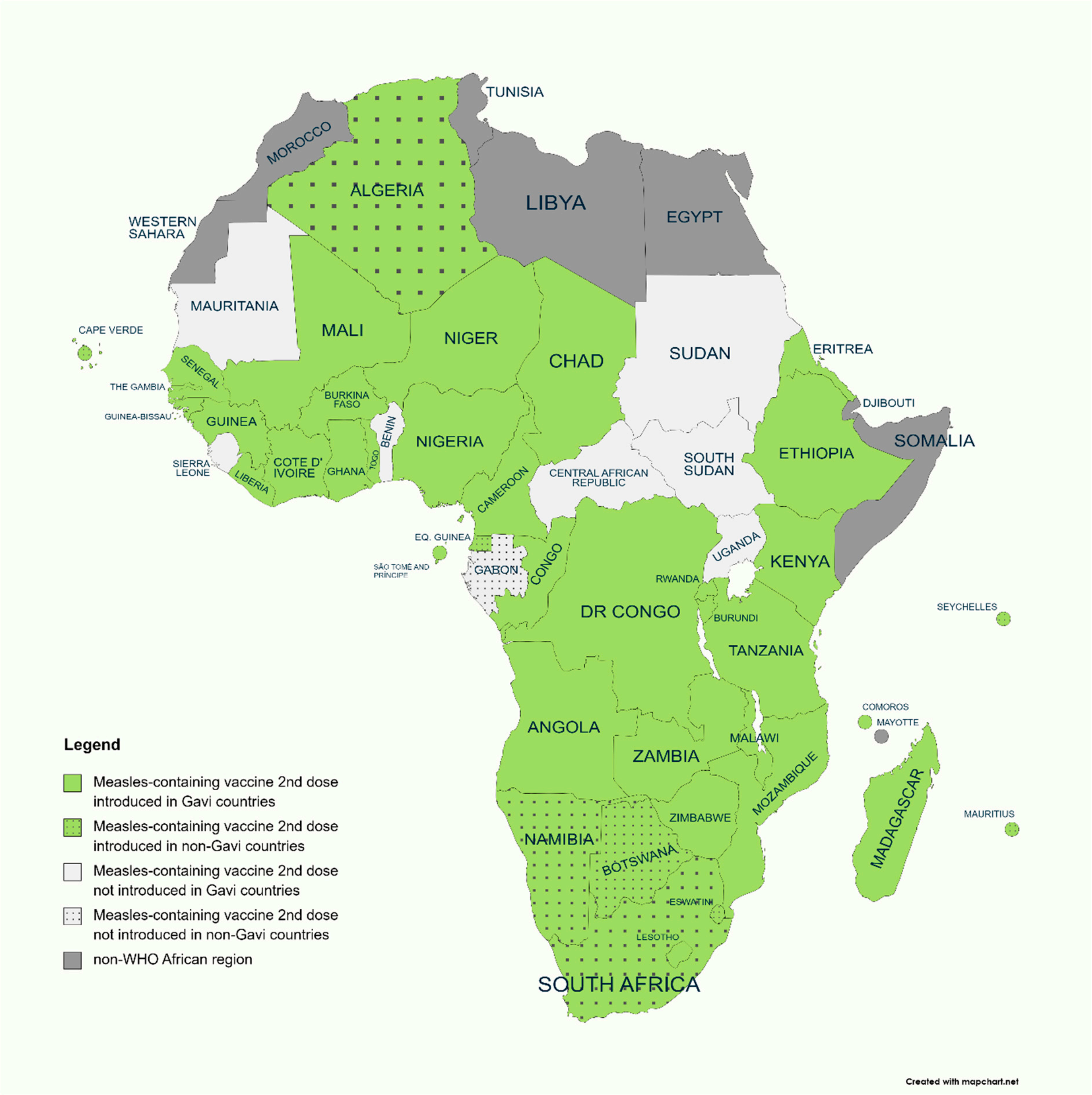

Figure S4. Status of MCV2 introduction in the WHO African region as of May 2022.

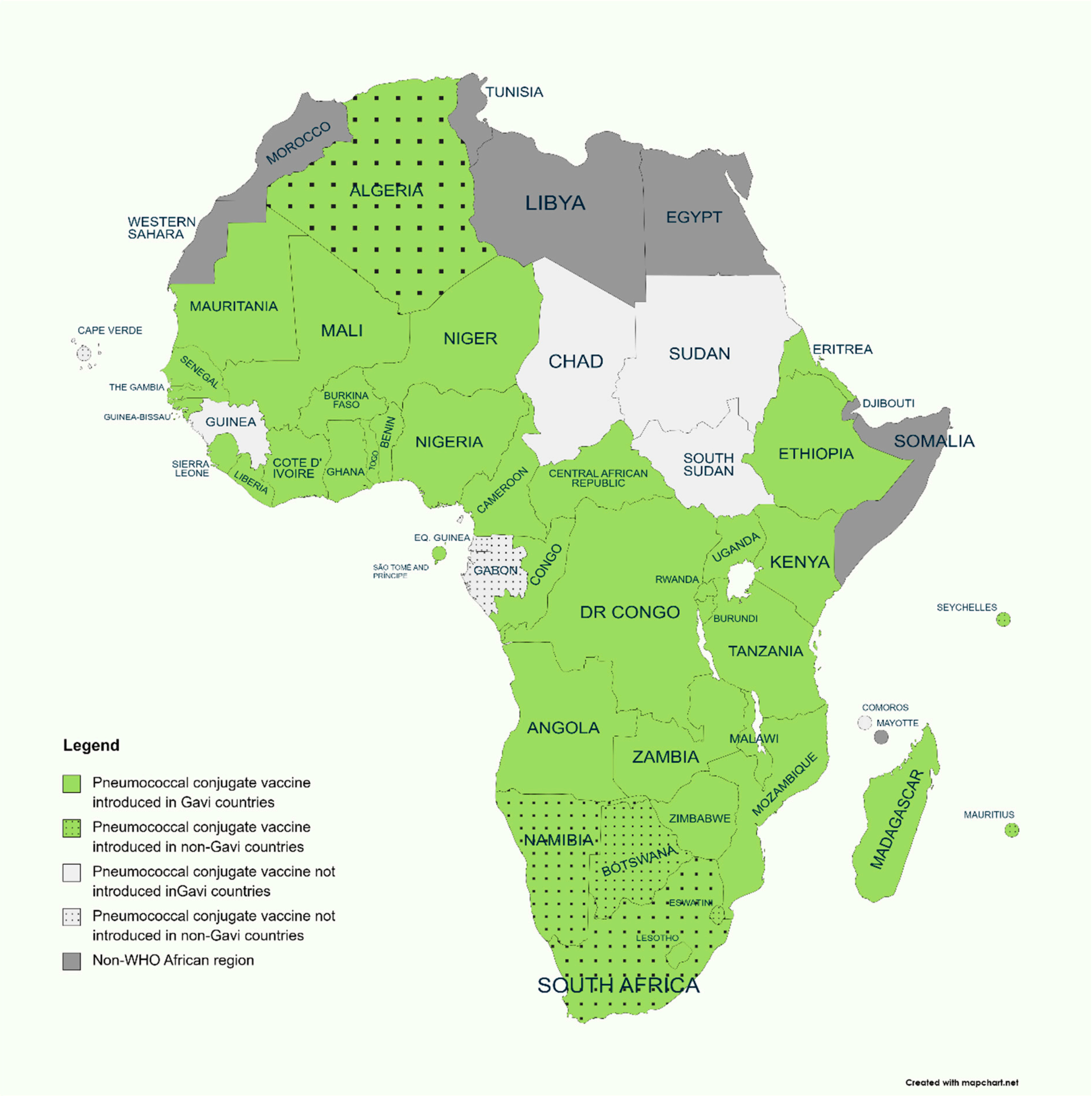

Figure S5. Status of PCV introduction in the WHO African region as of May 2022.

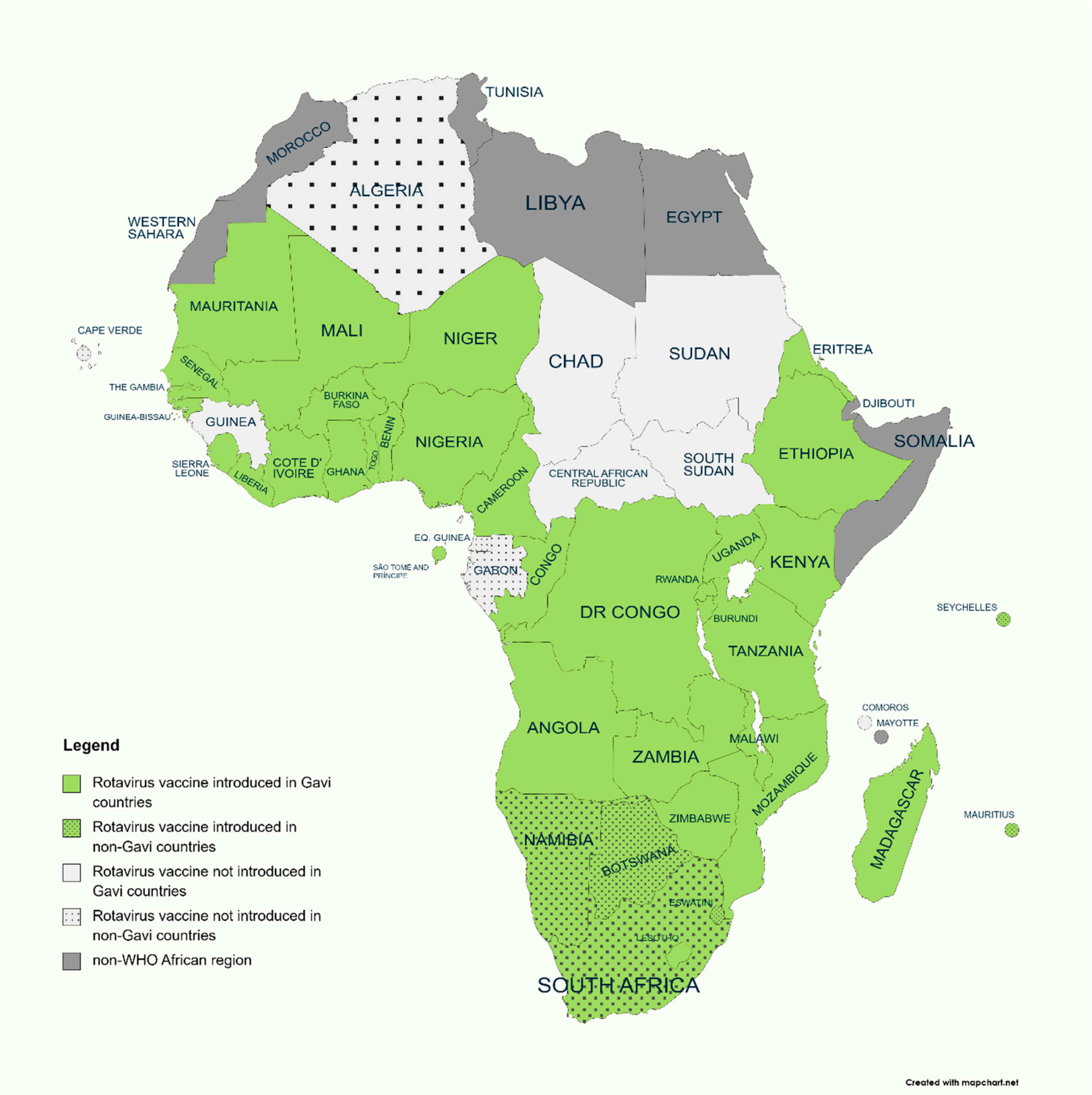

Figure S6. Status of rotavirus vaccine introduction in the WHO African region as of May 2022.

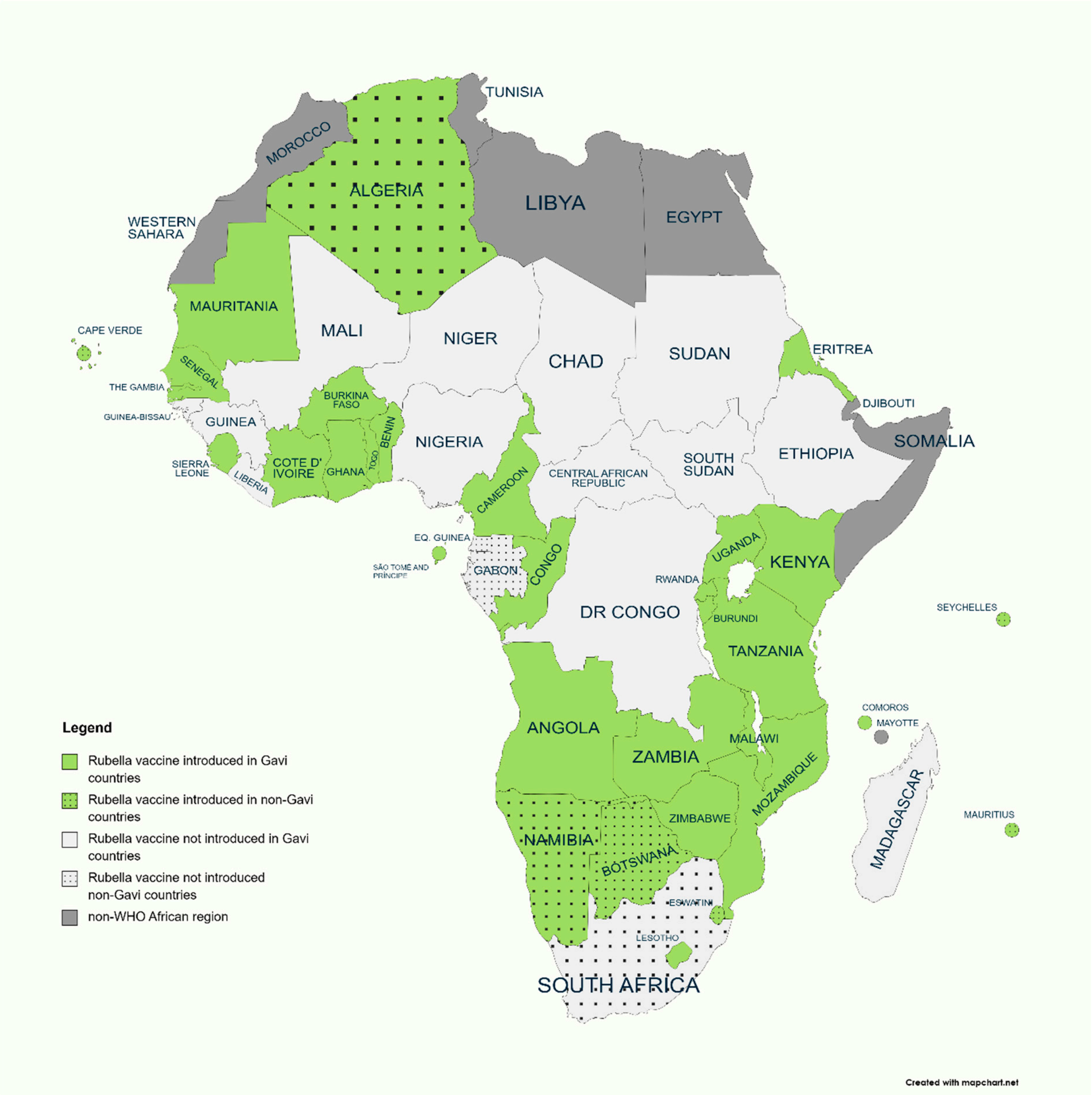

Figure S7. Status of rubella vaccine introduction in the WHO African region as of May 2022.

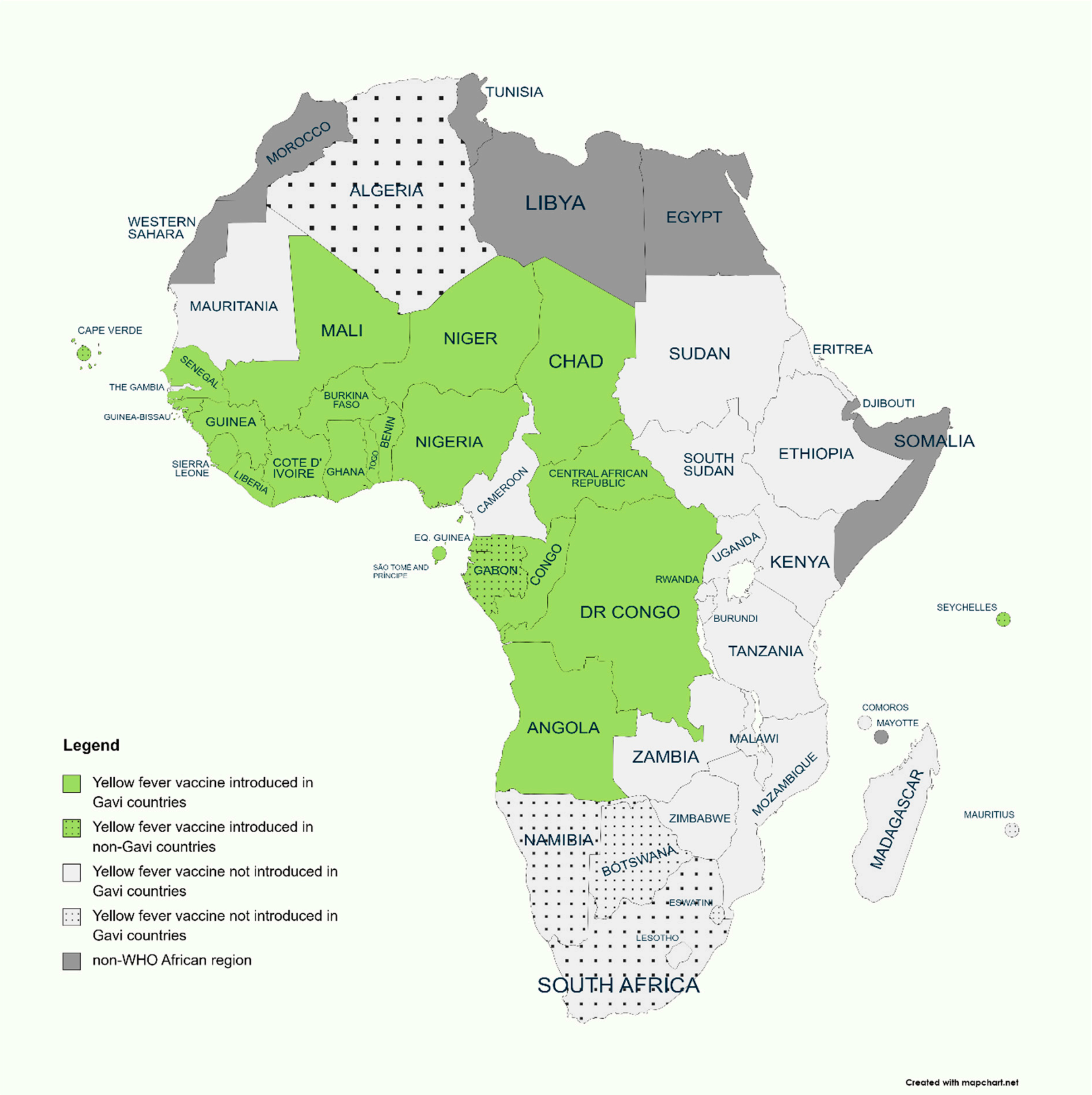

Figure S8. Status of yellow fever vaccine introduction in the WHO African region as of May 2022.

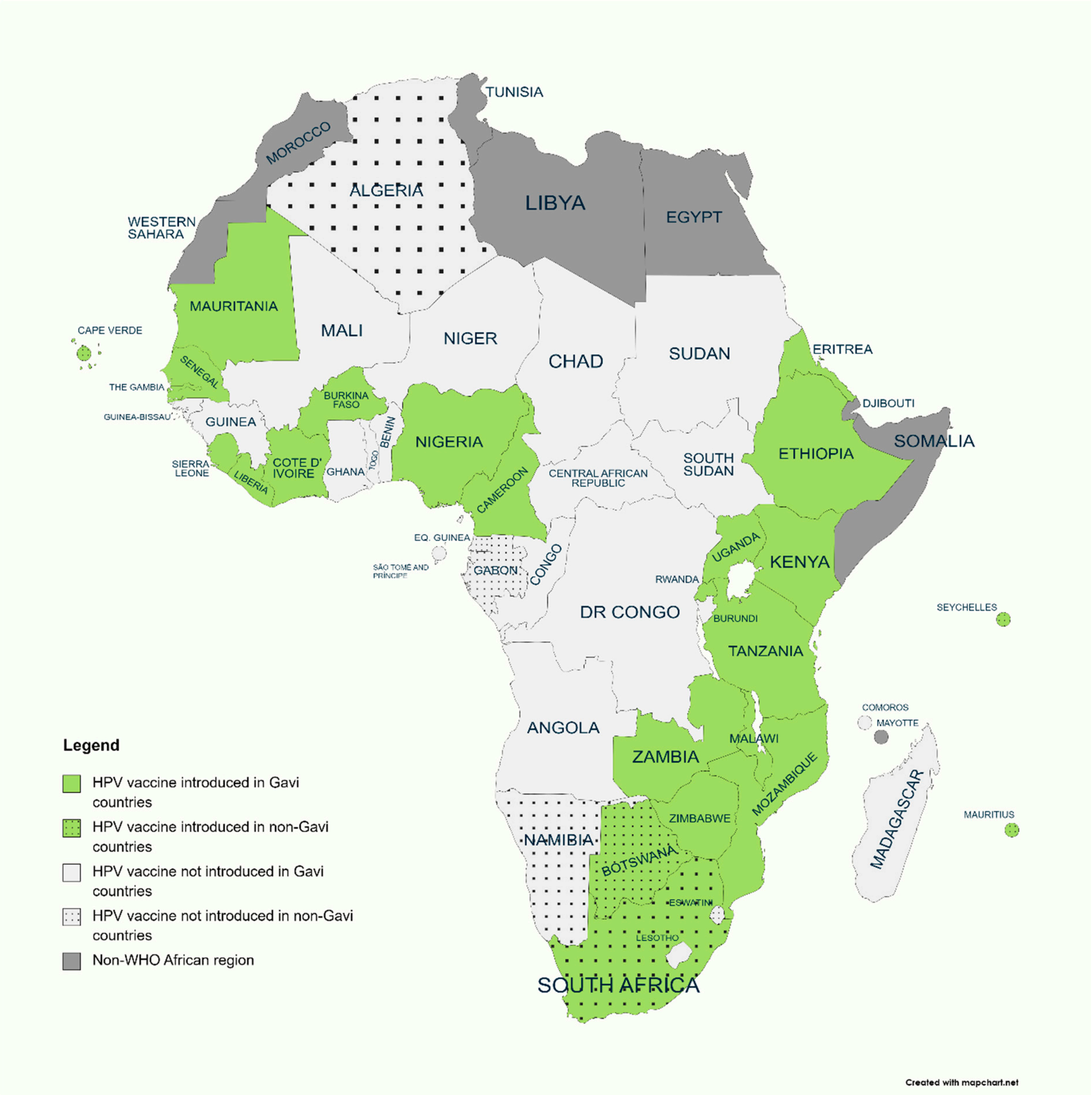

Figure S9. Status of HPV vaccine introduction in the WHO African region as of May 2022*

(*HPV has just been introduced in Nigeria as of October 2023).

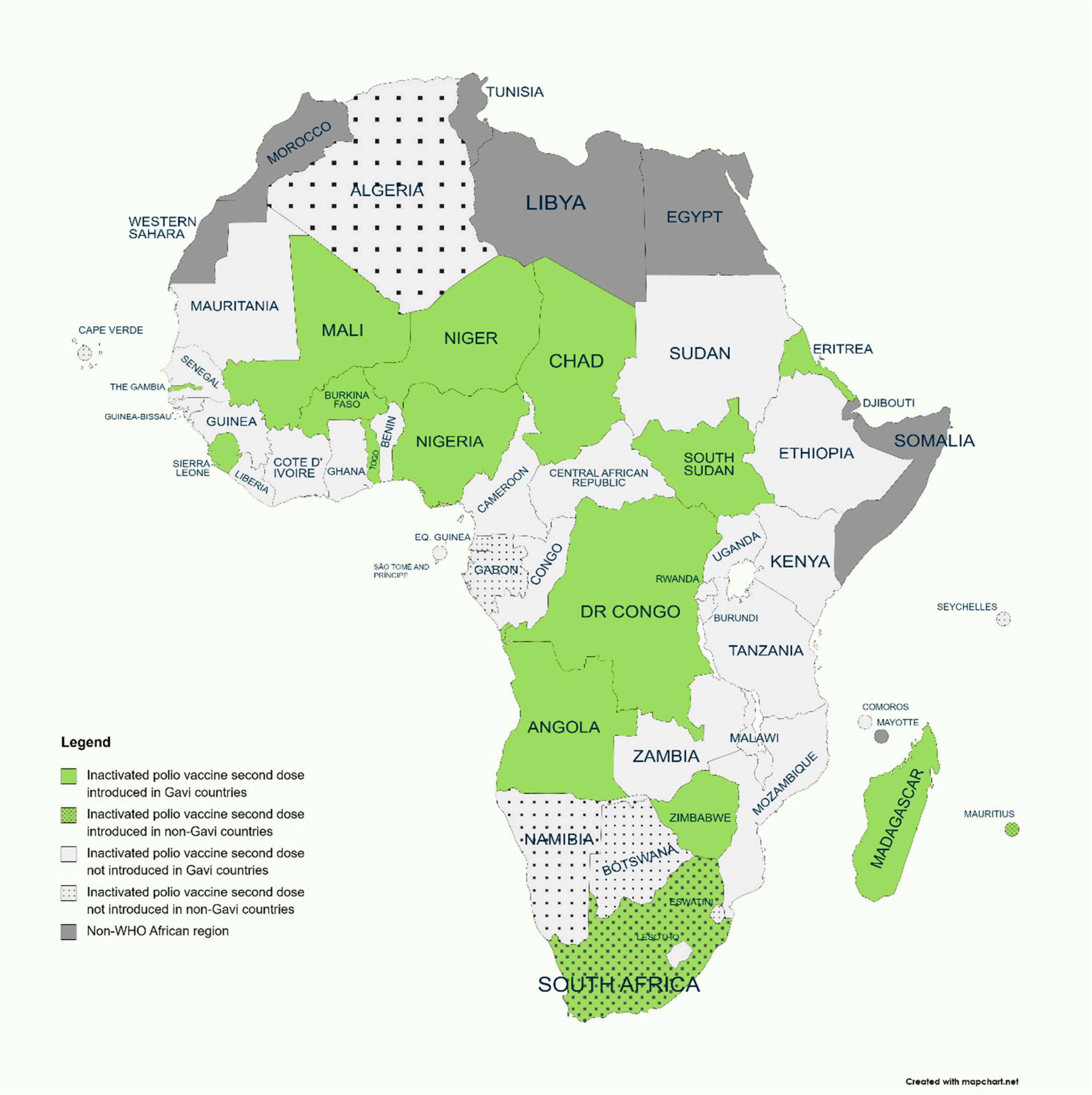

Figure S10. Status of IPV2 introduction in the WHO African region as of May 2022.

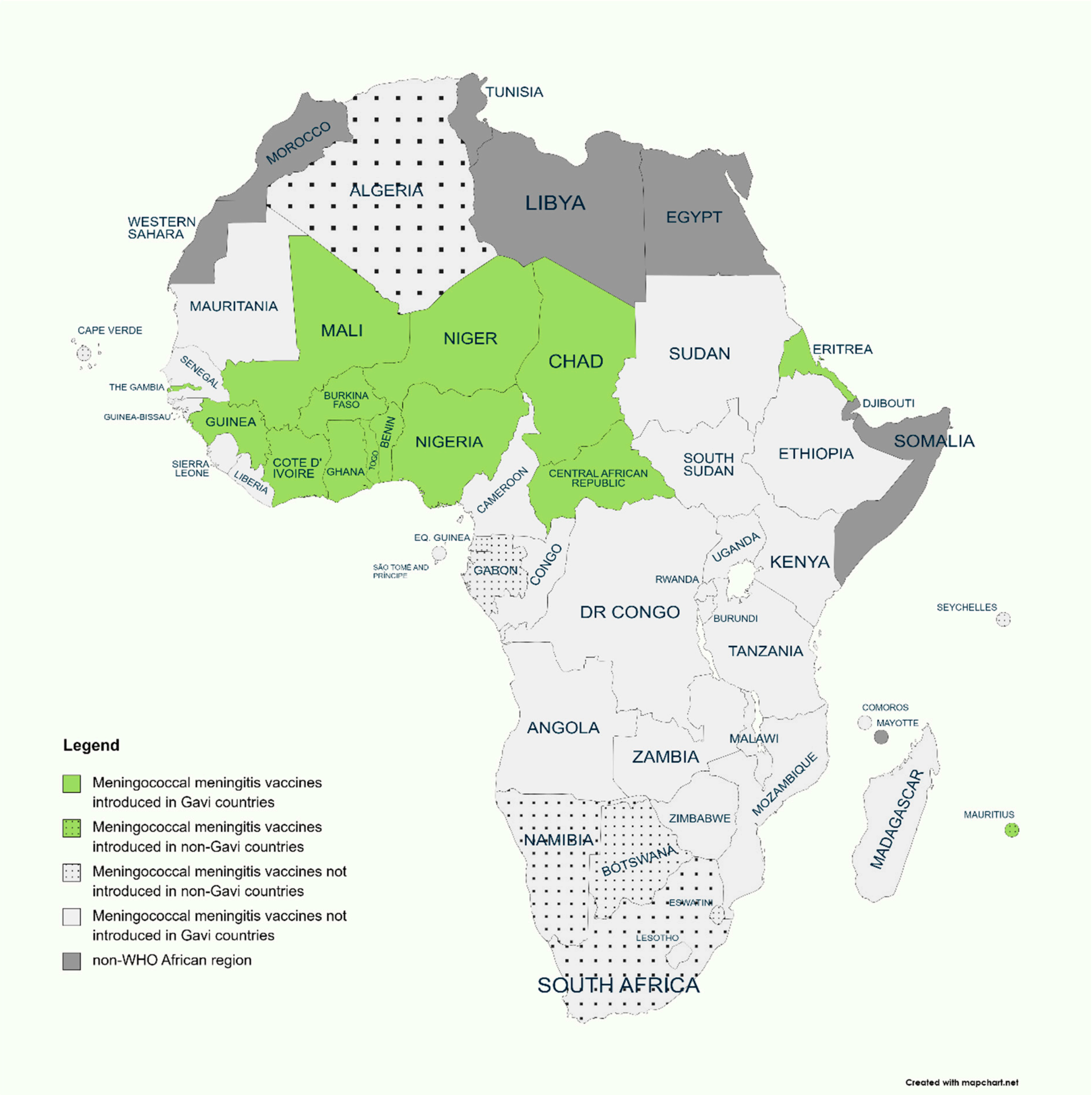

Figure S11. Status of meningococcal meningitis vaccine introduction in the WHO African region as of May 2022.

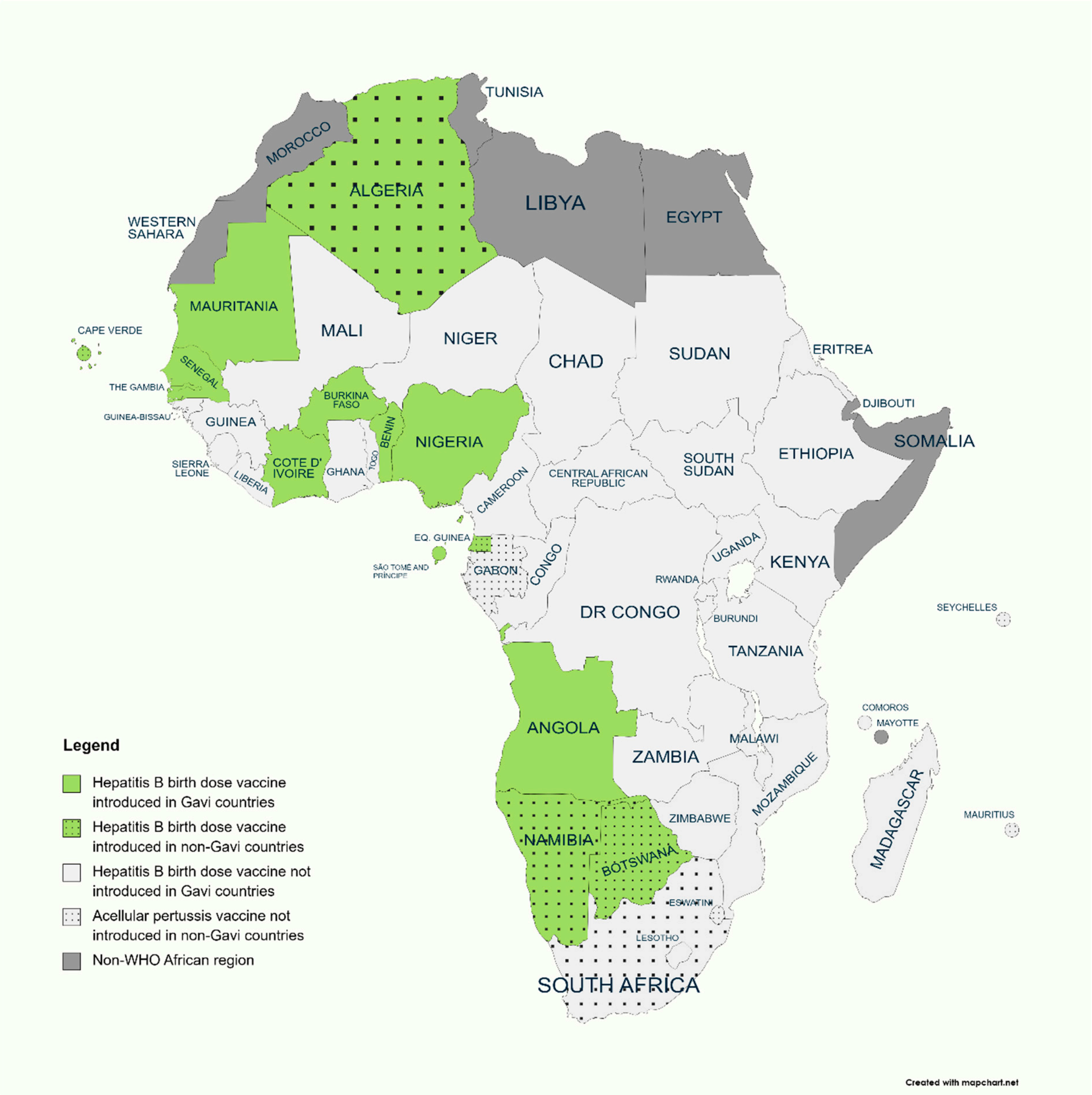

Figure S12. Status of HepB birth dose vaccine introduction in the WHO African region as of May 2022.

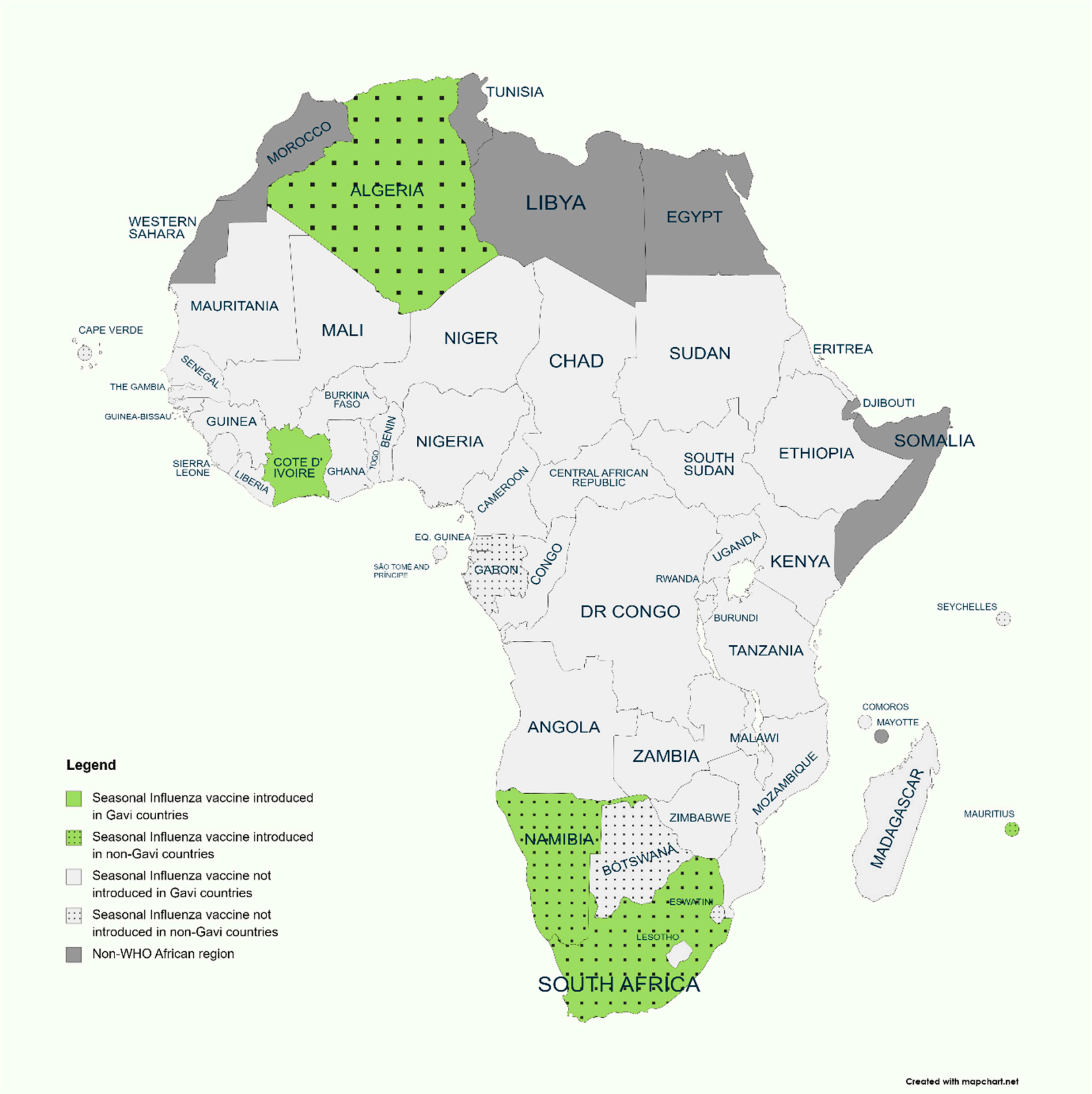

Figure S13. Status of seasonal influenza vaccine introduction in the WHO African region as of May 2022.

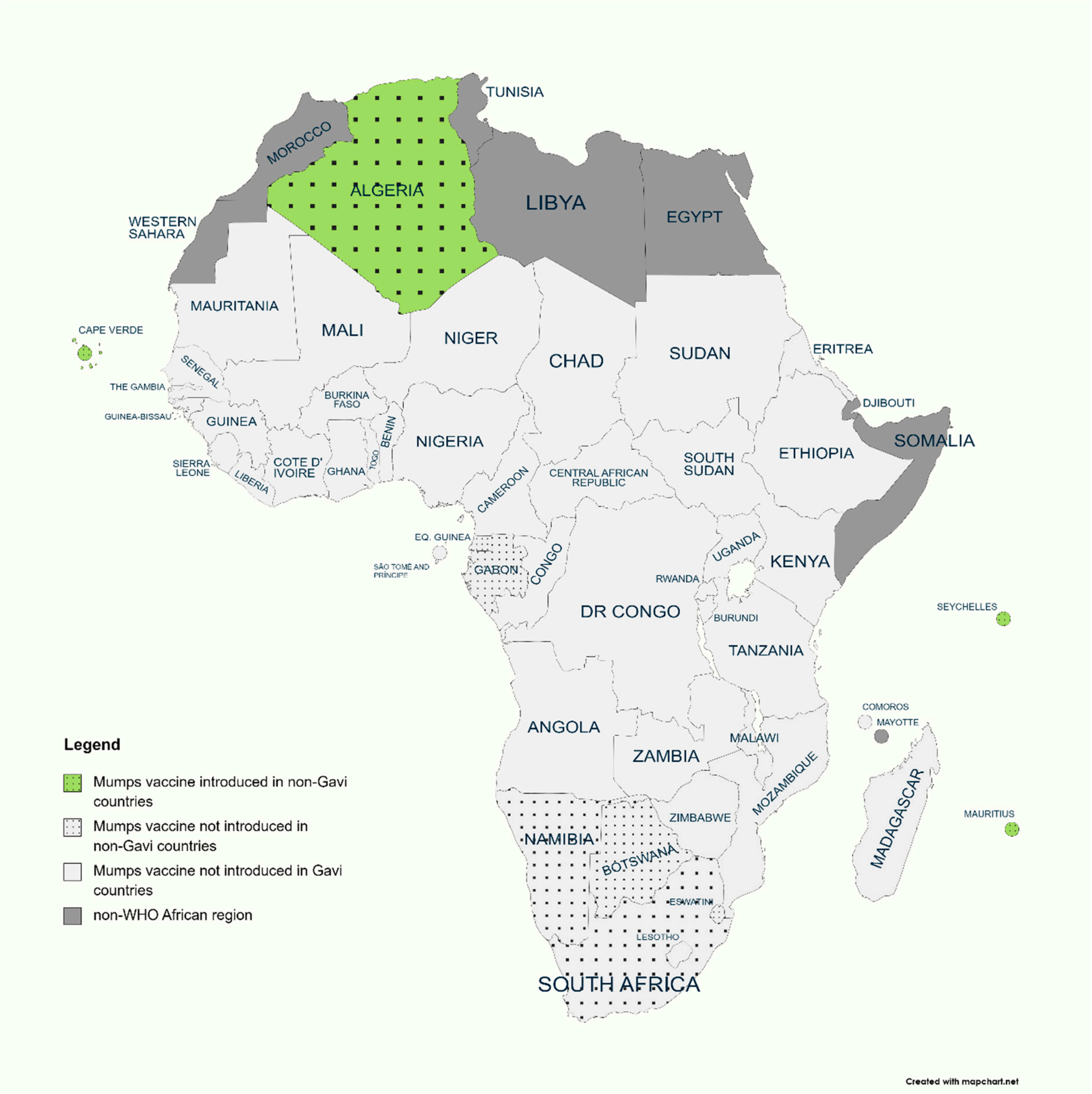

Figure S14. Status of mumps vaccine introduction in the WHO African region as of May 2022.

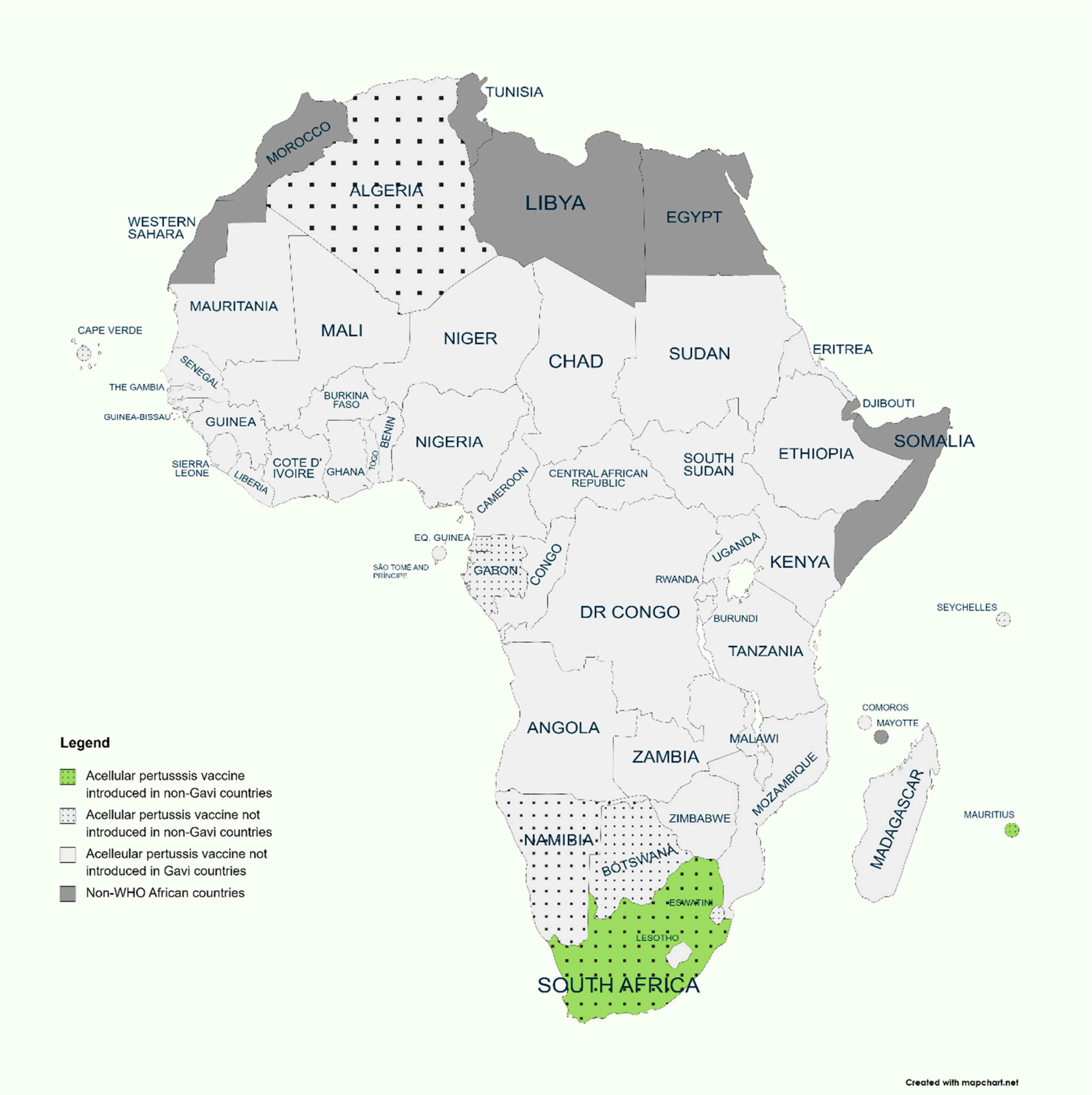

Figure S15. Status of acellular vaccine introduction in the WHO African region as of May 2022.

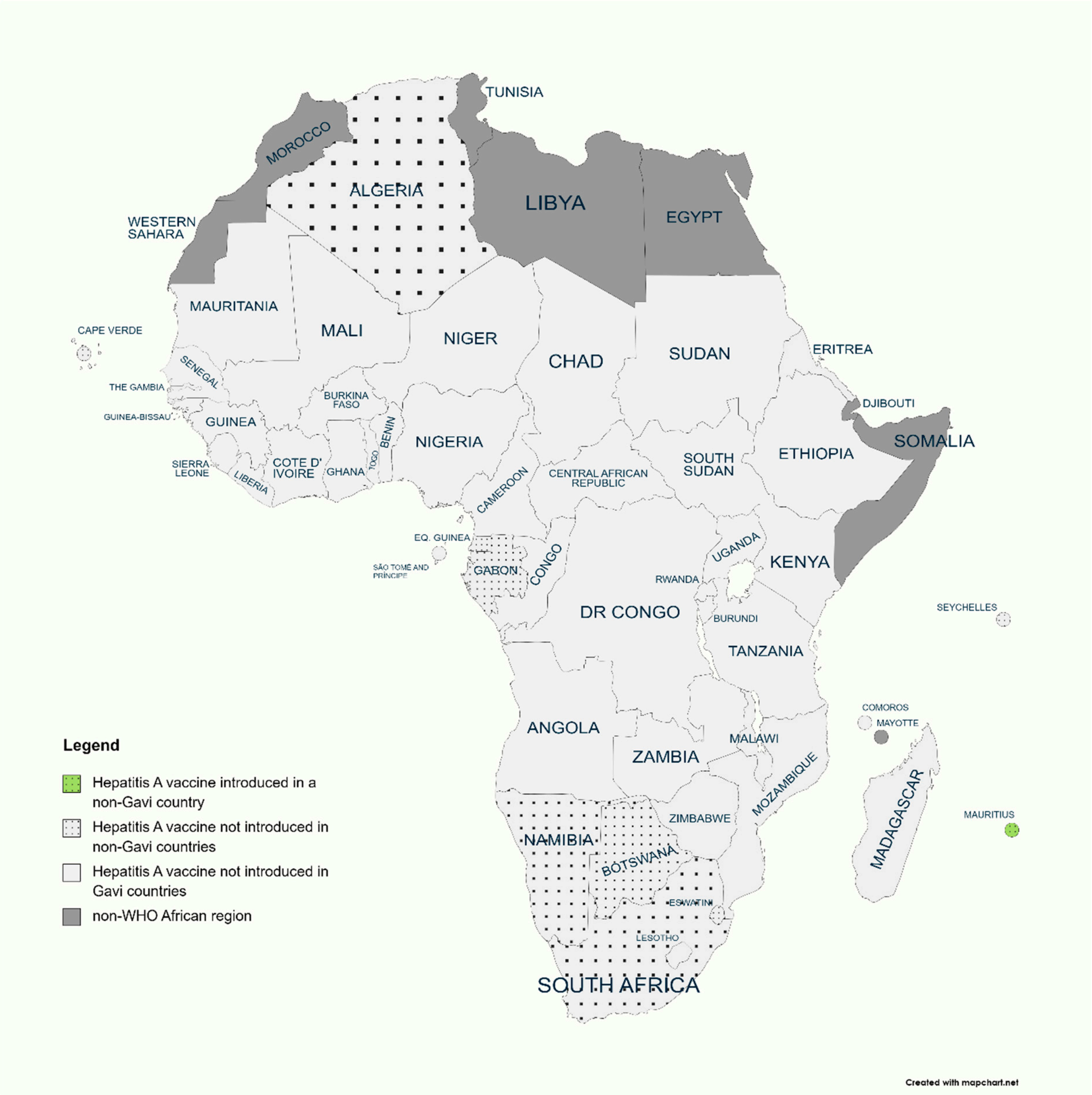

Figure S16. Status of hepatitis A vaccine introduction in the WHO African region as of May 2022.
