# Supplementary material for: New Vaccine Introductions in WHO African Region between 2000 and 2022
Source: Vaccines (Basel). 2023 Nov 16;11(11):1722. doi: 10.3390/vaccines11111722 (PMC10675678; doi:10.3390/vaccines11111722)
Supplement: Supplementary file 1 [file vaccines-11-01722-s001.zip › vaccines-2651925-supplementary table.pdf]

Supplementary Table S1. Summarises the number of Gavi eligible and ineligible countries that have introduced each new vaccine.

Vaccines	Gavi eligible (n = 37)	Gavi ineligible (n=10)
aP (acellular pertussis) vaccine	0	0
Hepatitis A vaccine	0	1
Hepatitis B vaccine	37	10
HepB birth dose	9	5
Hib (Haemophilus influenzae type B) vaccine	37	10
HPV (Human Papilloma Virus) vaccine	18	5
IPV (Inactivated polio vaccine)	37	10
IPV (Inactivated polio vaccine) 2nd dose	14	2
Measles-containing vaccine 2nd dose	32	9
Meningococcal meningitis vaccines (all strains)	13	1
Mumps vaccine	0	4
PCV (Pneumococcal conjugate vaccine)	33	7
Rotavirus vaccine	32	6
Rubella vaccine	25	7
Seasonal Influenza vaccine	1	4
YF (Yellow fever) vaccine	21	4
Japanese encephalitis	0	0
